# Supplementary material for: Pharmaceutical Company Targets and Strategies to Address Climate Change: Content Analysis of Public Reports from 20 Pharmaceutical Companies
Source: Int J Environ Res Public Health. 2023 Feb 11;20(4):3206. doi: 10.3390/ijerph20043206 (PMC9967855; doi:10.3390/ijerph20043206)
Supplement: Supplementary file 1 [file ijerph-20-03206-s001.zip › ijerph-2081817-supplementary.pdf]

Pharmaceutical Company Targets and Strategies to Address Climate Change:

Content Analysis of Public Reports from 20 Pharmaceutical Companies

Supplementary Material

Supplementary Table S1: Data Extraction Form

**Company information**	Name of company
	Headquarters
	2020 Annual revenue (Billion USD)
	Number of employees
	Ownership structure (public/ private)
	Lead modality
	Lead therapy area
**GHG emission reporting (latest reported value, baseline value and year of reporting) and reporting standards**	Scope 1 GHG emissions (Direct emissions related to on-site fuel combustion or fleet vehicles in metric tonnes carbon dioxide equivalent [Mt CO2e])
	Scope 2 GHG emissions, location-based (Indirect emissions related to purchased energy and fuel. Location based reflects the average emission intensity of grids on which energy consumption occurs in Mt CO2e)
	Scope 3 GHG emissions (Indirect emissions related to purchased goods, capital goods, fuel and energy related activities, upstream transportation and distribution, waste generated in operations, business travel, employee commuting, downstream transportation and distribution, processing of sold products, use of sold products, end-of-life treatment of sold products, leased assets, franchises, investments)
	Standards/ frameworks used to report GHG emissions
**GHG emission reduction targets**	Target period (baseline year and year company aims to achieve target)
	Target set (e.g., carbon neutral, net zero or percentage GHG emission reduction)
	International treaties, policies or initiatives targets are aligned with
**GHG emission reduction initiatives/ strategies**	What is the initiative/ strategy
	Which GHG emission scope will the initiative/ strategy likely address
	Which companies are implementing this strategy

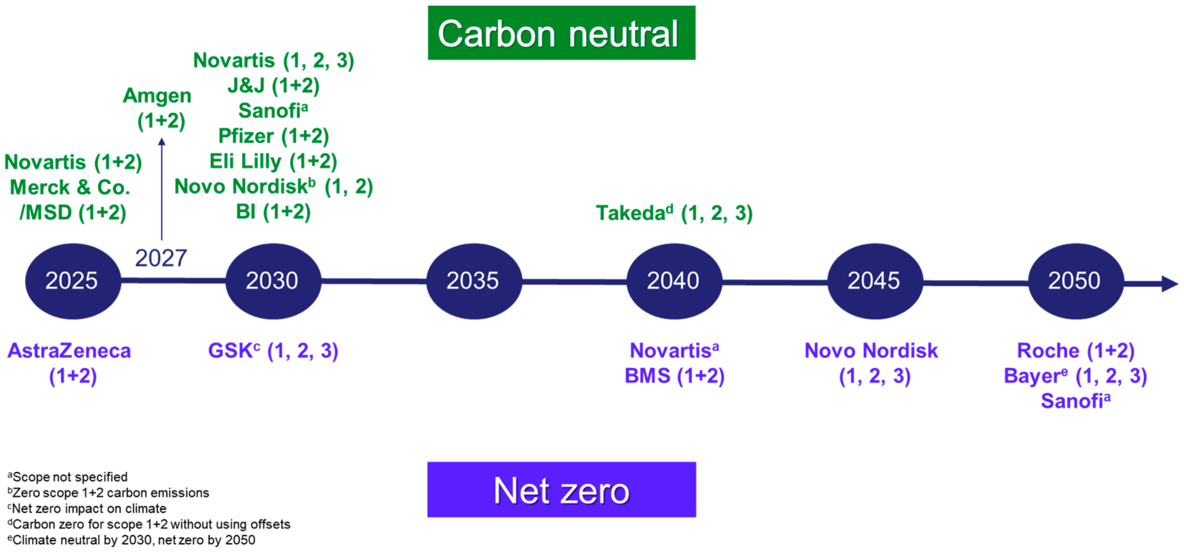

Supplementary Figure S1: Current reported company targets of carbon neutrality or net zero across scope 1, 2, and 3 (in brackets)

Supplementary Table S2: Current reported climate change targets of pharmaceutical companies

Company	Carbon Neutral (Baseline Year/ Target Year)	Net Zero (Baseline Year/ Target Year)	Percentage GHG Emission Reduction (Baseline Year/ Target Year)
**Roche**		Reduction in scope 1+2 GHG emissions to zero (2020/ 2050)	40% reduction in scope 1 + 2 GHG emissions (2020/ 2025)<br>18% reduction in scope 3 GHG emissions (2020/ 2025)<br>100% reduction in scope 1+2 GHG emissions (2025/2050)
**Novartis**	Carbon neutral in scope 1+2 operations (2016/ 2025)<br><br>Carbon neutral across all scopes (2016/ 2030)	Net zero (scope not specified)<br>(2016/ 2040)	100% reduction in scope 1+2 GHG emissions (2016/ 2025)<br>50% reduction in scope 1, 2 and 3 (2016/ 2030)
**AbbVie**			25% reduction in scope 1 + 2 GHG emissions (2019/ 2025)<br>50% reduction in scope 1+2 GHG emissions (2019/ 2035)
**Johnson & Johnson (J&J)**	Carbon neutral in scope 1+2 (2016/ 2030)		60% reduction in scope 1+2 (2016/ 2030)<br>20% reduction in scope 3 (2016/ 2030)
**Bristol Myers Squibb (BMS)**		Net zero in scope 1+2 GHG emissions (2020/ 2040)	
**Merck & Co**	Carbon neutral in scope 1+2 (2019/ 2025)		40% reduction in scope 1+2 (2015/ 2025)<br>46% reduction in scope 1+2 GHG emissions (2019/ 2030)<br>30% reduction in value chain GHG emissions (2019/ 2030)
**Sanofi**	Carbon neutral (scope not specified)<br>(2019/2030)	Net zero (scope not specified)<br>(2019/2050)	50% reduction in scope 1+2 GHG emissions (2015/ 2025)<br>55% reduction in scope 1+2 GHG emissions (2019/ 2030)<br>14% reduction in value chain GHG emissions (2019/ 2030)
**Pfizer**	Carbon neutral across scope 1+2 (2019/ 2030)		46% reduction in scope 1+2 GHG emissions (2019/ 2030)
**GlaxoSmithKline (GSK)**		Net zero in scope 1, 2 and 3 (2016/ 2030)<br>Net zero impact on climate (2016/ 2030)<br>Net positive impact on nature (2016/2030)	20% reduction in scope 1+2 carbon emissions (2016/ 2030)<br>25% reduction in scope 3 carbon emissions (2016/2030)
**Takeda**	Carbon zero without using offsets for scope 1+2 (2019/ 2040)<br>Carbon neutral across scope 3 (2019/ 2040)		40% reduction in scope 1+2 GHG emissions (2016/ 2025)<br>15% reduction in scope 3 GHG emissions (2008/ 2025)<br>50% reduction in scope 3 GHG emissions (2019/ 2040)
**AstraZeneca**		Net zero without using offsets for scope 1+2 (2015/ 2025)	100% reduction in scope 1+2 GHG emissions (2015/ 2025)<br>20% reduction in selected scope 3 GHG emissions (2015/ 2025)
**Amgen**	Carbon neutral in scope 1+2 emissions<br>(2019/ 2027)		100% GHG reduction in scope 1+2 (2019/ 2027)
**Gilead Sciences**			25% reduction in scope 1+2 GHG emissions (2016/ 2025)

			46% reduction in scope 1+2 GHG emissions (2019/ 2030)<br>15% reduction in scope 3 GHG emissions (2019/ 2030)
**Eli Lilly**	Carbon neutral in scope 1+2<br>(?/ 2030)		
**Novo Nordisk**	Zero scope 1+2 carbon emissions (2020/ 2030)	Net zero across value chain (2020/ 2045)	100% reduction in scope 1+2 (2020/ 2030)<br>100% reduction in selected scope 3 (transport and distribution) (2020/ 2030)
**Bayer**		Net Zero emissions in entire value chain (2019/ 2050)	20% reduction in scope 1+2 (2019/ 2024)<br>6% reduction in scope 3 (2019/ 2024)<br>42% reduction in scope 1+2 GHG emissions (2019/ 2030)<br>12.3% reduction in scope 3 GHG emissions (2019/ 2030)
**Boehringer Ingelheim (BI)**	Carbon neutral in scope 1+2 (?/ 2030)		
**Astellas Pharma**			30% reduction in scope 1+2 GHG emissions (2015/ 2030)<br>20% reduction in scope 3 GHG emissions (2015/ 2030)
**Viatris**			
**Teva Pharmaceutical**			33% reduction in scope 1+2 GHG emissions (2017/ 2030)

Supplementary Table S3: International treaty, policy or initiative that companies report aligning their GHG emission reduction targets with

International Treaty, Policy or Initiative	Definition of Treaty, Policy or Initiative	Number (Name of Companies that Align Climate Change Targets with Treaty, Policy or Initiative)
**United Nations (UN) Sustainability Development Goals**	Seventeen shared global goals for creating a better future, including tackling climate change (SDG 13), culminating in 2030	20 (Roche, Novartis, AbbVie, J&J, BMS, Merck & Co, Sanofi, Pfizer, GSK, Takeda, AstraZeneca, Amgen, Gilead Sciences, Eli Lilly, Novo Nordisk, Bayer, BI, Astellas, Viatris, Teva)
**UN Global Compact**	Special UN pact and world's largest CSR initiative, calling on companies to align their operations and strategies with Ten Principles, including environmental principles (7-9)	19 (Roche, Novartis, J&J, BMS, Merck & Co, Sanofi, Pfizer, GSK, Takeda, AstraZeneca, Amgen, Gilead, Eli Lilly, Novo Nordisk, Bayer, BI, Astellas, Viatris, Teva)
**Paris Agreement**	International treaty adopted in 2015 with the goal to keep the rise in mean global temperature to below 2°C, preferably limited to 1.5°C, and the need to reduce emissions by 50% by 2030 in order to meet this goal	18 (Roche, Novartis, AbbVie, J&J, Merck & Co, Pfizer, GSK, Takeda, AstraZeneca, Amgen, Gilead Sciences, Eli Lilly, Novo Nordisk, Bayer, BI, Astellas, Viatris, Teva)
**IPCC**	UN body developed to assess the science related to climate change and develop recommendations for GHG emissions reductions to keep the rise in mean global temperature to below 2°C, preferably limited to 1.5°C	10 (Novartis, J&J, Pfizer, GSK, Takeda, Amgen, Eli Lilly, Novo Nordisk, Bayer, Astellas,)
**Science Based Targets Initiative (SBTi)**	Collaboration between CDP, UN Global Compact, World Resources Institute and World-Wide Fund for Nature to help companies set emission reduction targets in line with climate science and Paris Agreement goals	16 (Novartis, AbbVie, J&J, BMS, Merck & Co, Sanofi, Pfizer, GSK, Takeda, AstraZeneca, Gilead Sciences, Novo Nordisk, Bayer, Astellas, Viatris Teva)
**Race to Zero (Business Ambition for 1.5°C)**	Led by the SBTi, in partnership with UN Global Compact calling on companies to commit to science-based emissions reduction targets in line with a 1.5°C future	9 (Novartis, AbbVie, J&J, Sanofi, Pfizer, GSK, Takeda, AstraZeneca, Bayer)
**2°C Scenario of the International Energy Agency**	International Energy Agency's report on targets and strategies to keep	1 (Astellas)

	mean global temperature rise limited to 2°C	
**European Union Green Deal**	Set of policy initiatives with the aim to reduce emissions by 50% by 2030 and make the EU climate neutral by 2050	1 (Bayer)

Supplementary Table S4: Scope 1, 2, and 3 GHG emissions values (Mt CO_2e) and change from baseline year of reporting

(Arrows indicate direction of change of GHG emissions over the reporting period, either increased or decreased; Text in red highlights increase in GHG emissions))

Company Name (Reporting Period)	Scope 1 (Mt CO2e)[1]	Scope 2 (Mt CO2e)[1]	Scope 3	
			Scope 3[2] (Mt CO2e)[1]	Reported scope 3 emission categories
**Roche (2017-2020)**	256 815 (↓ 13.0%)	189 003 (↓ 30.0%)	56 937 (↓ 72.1%)	Indirect emissions (not included in scope 2) in the value chain; business flights
**Novartis (2016-2020)**	378 200 (↓ 18.5%)	396 900[3] (↓ 34.9%)	25 100 (↓ 80.0%)	Business travel
**AbbVie (2016-2020)**	265 000 (↓ 13.1%)	243 000 (↓ 20.3%)	-	Not included
**J&J (2018-2020)**	363 924 (↓ 19.6%)	641 489[3] (↓ 5.9%)	20 373 117 (↑ 3.3%)	Purchased goods and services; Upstream transportation and distribution; Business travel; Employee commuting; Capital goods; Fuel- and energy-related activities; Upstream leased assets; Waste generated in operations; Use of sold products (direct and indirect); End of life treatment of sold products (*Data for Downstream Transportation and Distribution not available for 2020)
**Bristol Myers Squibb (2015-2020)**	165 340 (↓ 21.5%)	112 700 (↓ 25.3%)	11 890 (↓ 82.9%)	Business Air Travel
**Merck & Co. (2015-2020)**	738 400 (↓ 15.0%)	392 400[3] (↓28.4%)	7 103 450 (↑ 27.2%)	Purchased goods and services, Capital Goods, GHG emissions from fuel and energy-related activities not included in Scopes 1 & 2, Upstream transportation and distribution, Waste generated in operations (excluding recycled & composted waste), GHG emissions related to employee business travel, Employee commuting, Downstream transportation and distribution, GHG emissions from use of sold products, End-of-life treatment of sold products
**Sanofi**	467 497 (↓ 14.3%)	232 117 (↓ 44.6)	5 039 775 (↓ 9.3%)	Purchased goods, capital goods, Fuel and energy related

**(2015-2020 – scope 1+2<br>2019-2020 – scope 3)**				activities, Upstream transportation and distribution, Waste generated in operations, Business travel, Employee commuting, Downstream transportation and distribution, Processing of sold products, Use of sold products, End-of-life treatment of sold products
**Pfizer<br>(2012-2020)**	690 000<br>(↓ 31%)	660 000<br>(↓ 33%)	-	Not included
**GlaxoSmithKline<br>(2017-2020)**	773 000<br>(↓ 13.3%)	507 000[3]<br>(↓ 16.2%)	14 620 000<br>(↓ 19.5%)	Purchased goods and services, Capital goods, Fuel and energy related activities, Transportation and distribution (upstream), Waste generated in operations, Business travel, Employee commuting, Leased assets (upstream), Transportation and distribution (downstream), Processing of sold products, Use of sold products (Emissions from use of propellant-based inhalers by patients), End of life, Leased assets (downstream), Franchises, Investments
**Takeda<br>(2016-2019 – scope 1+2<br>2018-2019 – scope 3)**	333 000<br>(↔)	319 000[3]	3 943 000<br>(↑ 2%)	Purchased goods and services, Fuel and energy related activities not included in Scope 1 and 2, Upstream transportation & distribution, Business travel, Employee commuting, all other applicable categories
**AstraZeneca<br>(2015-2020)**	224 771<br>(↓ 23.9%)	212 003[3]<br>(↓ 23.4%)	7 803 145<br>(↑ 3.5%)	Purchased goods and services, Capital goods, Fuel and energy related, Upstream transportation and distribution, Waste generated in operations, Business travel, Employee commuting, Upstream leased assets, Downstream transportation and distribution, Processing of sold products, Use of sold products, End of life treatment of sold products, Downstream leased assets, Franchises, Investments
**Amgen**	107 000<br>(↓ 15.1%)	136 000<br>(↓ 53.1%)	2 626 600<br>(↓ 8.0%)	Purchased Goods and Services, Capital Goods, Fuel- and

**(2007-2020 – scope 1+2 2018 – scope 3)**				Energy-Related Activities, Upstream Transportation & Distribution, Waste Generated in Operations, Business Travel, Employee Commuting
**Gilead Sciences (2016-2019)**	52019 (↑ 16.2%)	42 546[3] (↓ 8.6%)	-	Not included
**Eli Lilly (2012-2020)**	158 650 (↓ 27.9%)	550 922 (↓26.1%)	176 000 (↓ 33.8%)	Employee business travel (personal and rental cars, taxi, rail, and air travel); Employee commuting; Contracted product transportation and distribution; Waste generated in operations No additional scope 3 emissions from remote working were included. Scope 3 Emissions do not include non-Kyoto compounds. Scope 3 data does not include emissions from sales force travel using company vehicles, use of Lilly owned aircraft, on-site waste incineration, or product distribution with Lilly owned vehicles, as these are included in the Scope 1 data.
**Novo Nordisk[4] (2014-2021)**	76 829 (↓ 30.4%)	128 625[3] (↓ 32.8%)	1 449 009	Purchased goods and services; Capital goods; Fuel and energy related activities (not included in scope 1+2); Upstream transportation and distribution; Waste generated in operations; Business travel; Employee commuting;
**Bayer[5] (2019-2020)**	2 010 000 (↓ 3.4%)	1 750 000[3] (↓ 1.2%)	8 860 000 (↓ 11.8%)	Purchased goods and services; Capital goods; Fuel- and energy-related activities; Upstream transportation and distribution; Waste; Business travel; Employee commuting; End-of-life treatment of sold products.
**Boehringer Ingelheim (2010-2020)**	253 104 (↓ 18.6%)	159 664 (↓ 34.1%)	-	Not included
**Astellas Pharma (2015-2020)**	57 403 (↓ 35.7%)	53 814 (↓ 52.3%)	176 369 (↓ 28.3%)	Purchased goods and services; Capital goods; Fuel and energy related activities not included in scope 1 and 2; Transportation and distribution; Waste generated

				in operation; Business travel (by airplane); Employee commuting; Use of sold products; End-of-life treatment of sold products
**Viatris (2015-2020)**	311 000 (↑ 2.9%)	348 000 (↓ 1.7%)	-	Not included
**Teva (2016-2020)**	296 347 (↓16.7%)	335 674 (↓ 18.7%)	-	Not included
**% Reported**	100%	100%	70%	
**% Improved (of reported)**	85%	95%	64%	
**Median**	280 673	281 000	3 284 800	
**Total**	7 978 299	7 410 857	72 306 912	

[1] Percentage change from baseline year and direction of change reported in brackets
[2] Reported scope 3 definitions vary
[3] Where both market- and location-based scope 2 is reported, location-based is used in the table
[4] Scope 1, 2 and 3 data available on the Carbon Disclosure website https://www.cdp.net/en/responses?utf8=%E2%9C%93&queries%5Bname%5D=novo+nordisk
[5] Bayer data includes data on their crop science, pharmaceuticals, consumer health and other portfolios
